# Pain-attributed care task difficulty among dementia caregivers with chronic pain

**DOI:** 10.3389/fpain.2025.1661457

**Published:** 2025-11-05

**Authors:** Shelbie G. Turner, Aryn Lee, Karl A. Pillemer, M. Carrington Reid

**Affiliations:** ^1^ Division of Geriatrics and Palliative Medicine, Weill Cornell Medicine, New York, NY, United States; ^2^ College of Human Ecology, Cornell University, Ithaca, NY, United States

**Keywords:** caregiving, chronic pain, activities of daily living, racial differences, caregiving difficulty

## Abstract

**Introduction:**

Chronic pain is highly prevalent among dementia family caregivers (henceforth “caregivers”). We used a nationwide sample of caregivers with chronic pain to identify the extent to which caregivers attribute pain to any difficulty they have with caregiving.

**Methods:**

Caregivers (*N* = 269) reported if they experienced difficulty performing ten individual care tasks and if ‘yes’, how much of the difficulty they attributed to pain (0 = not a reason for my difficulty, 10 = the biggest reason for my difficulty). We ran ANOVA models to determine between-group differences in pain-attributed difficulty with care tasks.

**Results:**

When asked about the extent to which pain contributed to the difficulty helping care recipients with a given care task, caregivers’ average response was 6.81 for basic activities of daily living and 6.49 for instrumental activities of daily living. Compared to White caregivers, Black caregivers attributed less of their difficulty with basic activities of daily living to pain (estimate = –1.17, *p* = 0.04).

**Discussion:**

Caregiver pain is not only highly prevalent may also be consequential to caregiving outcomes.

## Introduction

As the population continues to age and the prevalence of dementia rises, the demand on family caregivers continues to grow. Dementia family caregivers, many of whom are older adults themselves, are often challenged by managing their own health conditions alongside taking care of their relatives (1). The presence of these conditions and the time and energy required to manage them can negatively impact the quality of care caregivers provide to their relatives. Indeed, caregivers’ own health is associated with poor care recipient outcomes such as increased hospitalization (2, 3) and mortality rates (4, 5). Because of these factors, researchers and practitioners have focused attention on characterizing caregivers’ health and supporting caregivers’ ability to manage their own health while simultaneously performing their caregiving responsibilities.

Limited research or clinical guidance exists regarding the challenge pain poses to family caregivers. Pain is a highly prevalent health condition among family caregivers. Over 50% of family caregivers to older adults report bothersome pain and 40% report arthritis, one of the most common causes of chronic pain (6). Moreover, arthritis is the second most common chronic condition among caregivers behind high blood pressure (7). Caregiver pain is associated with care recipients’ unmet needs (8), but little is known about how pain interrupts or hinders the process of caregiving in ways that yield poor caregiving outcomes (9).

It is possible that poor caregiving outcomes result from the functional disability and physical discomfort associated with chronic pain among affected caregivers. Dementia family caregivers must often assist care recipients with basic activities of daily living (BADLs; bathing, dressing, toileting) and with instrumental activities of daily living (IADLs; grocery shopping, transportation). Assisting in these tasks while experiencing pain can make these tasks much more challenging to perform. However, to the best of our knowledge, no studies have characterized the difficulty caregivers with chronic pain experience when helping care recipients perform activities of daily living. Among caregivers who experience difficulty, knowing the extent to which pain is perceived to be the cause of that difficulty can illuminate the importance of this issue.

Accordingly, we sought to identify the extent to which dementia family caregivers with chronic pain perceive pain as a reason for any difficulty they have helping their care recipient perform ten common activities of daily living. We also characterized associations between pain-attributed difficulty performing care tasks and caregiver demographics (e.g., age, gender) and caregiving characteristics (e.g., hours of care per week). We hypothesized that, across all activities, caregivers would attribute much of their difficulty with care tasks to their pain. Specifically, we hypothesized that, on a scale from 0 (*not at all*) to 10 (*the biggest reason for my difficulty*), the average rating for each care task would be at least 5. We further hypothesized that caregivers who were older, female, from a racial/ethnic minoritized group, reported lower income, as well as those who reported greater caregiving intensity (e.g., more hours of care per week) would attribute more of their care task difficulty to pain. Indeed, research suggests that certain demographic characteristics (e.g., age, gender) are associated with greater pain-related functional disability both among the general population (10) and specifically among caregivers (6). Moreover, caregiving dynamics (e.g., hours of care provided each week) are associated with greater pain-related activity limitations (6).

## Methods

### Study procedure and participants

We conducted cross-sectional analyses using data from a nationwide survey of dementia family caregivers with chronic pain. Given limited available data on dementia family caregivers’ pain, we fielded a survey that focused specifically on pain's impact on caregivers’ health and caregiving outcomes. We partnered with Qualtrics to recruit the sample; Qualtrics maintains a proprietary service to garner online samples of research participants (i.e., market-research panels), which has been successfully used in behavioral science research (11).

In partnership with the Qualtrics team, we determined survey structure (e.g., length of completion) and sampling strategy (e.g., oversampling certain populations). We determined the target sample size of ∼250 caregivers via both statistical calculations of planned analyses and consideration of budgetary constraints. The sample was geographically representative based on U.S. census data, and we established a quota of at least 80 Black or Hispanic caregivers to support racial diversity in the sample given our interest in analyzing racial and ethnic differences. Additional details about the study design have been published previously (12).

Qualtrics sourced and recruited participants from pre-existing survey panels that they managed separately from our team. They determined the appropriate sampling structure based on our recruitment goals and completely managed the overall data collection process. Potentially eligible participants received an electronic invitation directly from Qualtrics to complete the survey, which included three screening questions to confirm eligibility. Eligibility criteria were as follows: caregiver experienced pain, aching, burning, or throbbing sensations on most days of every month for the past 3 months; caregiver's pain was not due to cancer; caregiver provided 20 or more hours of care per week to a relative/family member or friend with Alzheimer's disease or a related dementia.

Qualtrics fielded the survey on March 6th, 2024, and the survey closed on April 11th, 2024. The survey was designed to last no more than 15 min. Qualtrics delivered compensation to participants directly, which varied based on the participants’ pre-existing agreement with Qualtrics but averaged $7.50 per survey. Weill Cornell Medicine's Institutional Review Board deemed this study exempt from human subjects review given data were anonymous.

### Measures

#### Outcomes: pain-attributed difficulty with care tasks

Caregivers reported if the care recipient currently needed help with five individual BADLs: getting in and out of a bed or chair, eating, showering/bathing, dressing, and using the toilet; as well as five individual IADLs: grocery shopping, doing laundry, cleaning the home, making hot meals, and pursuing leisure activities. If the care recipient did need help with an activity, the caregiver then reported who was currently providing that help. If the caregiver reported that they were helping the care recipient to perform a given activity of daily living, they were then asked whether they had difficulty with that care activity. If caregivers endorsed experiencing difficulty helping a care recipient perform a given activity, caregivers were then asked: “*How much would you say your pain contributes to the difficulty you have helping the person you are caring for _____ [activity]?”* Responses on this item ranged from 0 (*not at all*) to 10 (*the biggest reason for my difficulty*). For caregivers reporting physical difficulty with at least one BADL, we averaged the extent to which pain contributed to that difficulty across all BADLs for which they reported difficulty. We repeated the same coding process for IADLs.

Of note, the questions about pain-attributed difficulty with care tasks were created by our research team in consultation with survey design experts because we were unable to identify any validated survey questions designed to measure pain-related functional impairment specifically in the context of caregiving. As such, these questions did not yet have established psychometric properties. To offer some psychometric data, we determined the items’ convergent validity by calculating Pearson's r between the 0-10 rating for pain-attributed difficulty (BADL and IADL) and the pain interference subscale of the PROMIS-29. The PROMIS-29 pain interference subscale was moderately positively correlated with the two pain-attributed care difficulty variables [BADL = .45 (*p* < .001) and IADL = .45 (*p* < .001)], suggesting that the single-item questions we created is a strong measure of pain-related difficulty performing various care tasks.

#### Predictors: caregiver demographics

We collected data on the following caregiving demographics: age, gender, race, ethnicity, and income. We also collected data on caregiving characteristics, including whether there were additional caregivers in the care network, the caregiver's relationship to the care recipient, how far the caregiver lived from the care recipient, how long the caregiver had been providing care, and how many hours per week the caregiver provided care.

### Analytic plan

We first determined the number of caregivers who helped their care recipient with one or more activities and, among those caregivers, the number who experienced difficulty performing one or more activities. Among the caregivers who reported difficulty, we computed the mean rating of how much pain contributed to that difficulty across all individual BADLs and IADLs. We also created a composite score representing each caregiver's mean pain-attributed difficulty across all five BADLs and all five IADLs. We then visually reviewed histograms and Q-Q plots of the composite pain-attributed BADL difficulty variable and the composite pain-attributed IADL difficulty variable and determined both were reasonably normally distributed and thus that parametric tests were appropriate to answer our research questions (visuals of histograms and Q-Q plots are available in Supplementary Materials S1). Thus, to assess for associations between pain-related difficulty with care tasks and caregiver demographics and caregiving characteristics, we computed an Analysis of Variance (ANOVA) model (via SAS PROC GLM with estimate and contrast commands) to determine between-group differences in the extent to which caregivers attributed difficulty with BADLs and IADLs to their pain. We computed one model, such that each estimate is adjusted for all other variables, and we used Scheffe's method to account for the testing of multiple comparisons in our model.

## Results

### Sample characteristics

**Figure 1:**
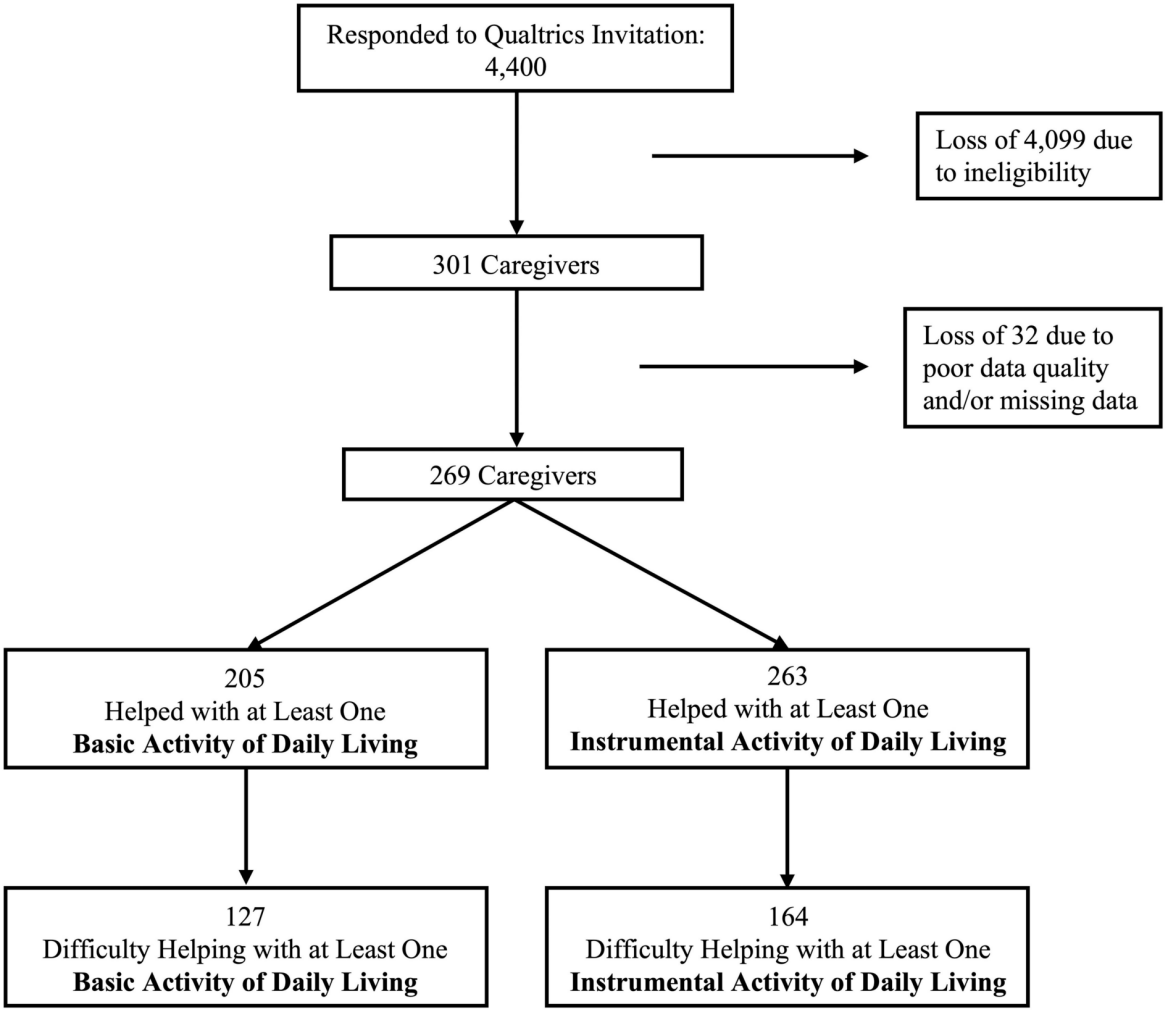
Analytic sample.

A total of 4,400 people responded to Qualtrics’ invitation to complete the survey, the vast majority of which (4,099) were ineligible because they did not meet eligibility criteria. We removed additional participants’ responses due to data quality issues (e.g., straight lining) or missing data, resulting in a final sample of 269 dementia family caregivers living with chronic pain (Figure 1).

Caregivers ranged in age from 19 to 83, with an average age of 45 years (SD = 12 years). They provided care for an average of 66 h per week (SD = 49 h; Range = 20–168 h). Additional sample characteristics are listed in Table 1.

**Table 1:** Sample characteristics.

Demographics and caregiving characteristics	Percentage (*N*)
Age	
18–39	35.96% (96)
40–59	49.06% (131)
60+	14.48% (40)
Gender	
Female	43.07% (115)
Male	56.18% (150)
Other	0.75% (2)
Race	
White	74.53% (199)
Black	16.85% (45)
Asian	5.62% (15)
Multiple races	3.00% (8)
Ethnicity	
Not Hispanic	68.54% (183)
Hispanic	31.46% (84)
Income	
Under $30,000	20.22% (54)
$30,000–$49,999	20.22% (54)
$50,000–$74,999	22.10% (59)
$75,000–$99,999	11.61% (31)
$100,000–$149,999	17.60% (47)
$150,000 or more	8.24% (22)
Relationship to care recipient	
Spouse/partner	13.48% (36)
Child or child-in-law	53.93% (144)
Related in another way	20.97% (56)
Unrelated friend	11.61% (31)
Presence of other caregivers	
No other caregivers	41.20% (11)
1+ other caregivers	58.80% (157)
Distance from care recipient	
Live in the same home	51.31 (137)
Less than 10 miles away	38.20 (102)
11+ miles away	10.49 (28)
Duration of caregiving	
Less than a year	9.36% (25)
1–2 years	46.44% (124)
3–5 years	31.46% (84)
6–10 years	6.37% (17)
More than 10 years	6.37% (17)
Hours of care per week	
20–39 h	39.33% (105)
40–59 h	22.10% (59)
60–79 h	11.24% (30)
80+ h	27.34% (73)

### Pain-attributed difficulty

Of the 269 caregivers, 205 helped their care recipient with at least one BADL. Of those 205 caregivers, 127 (62%) reported difficulty helping with at least one BADL (Figure 1). Among caregivers reporting difficulty helping with at least one BADL, the average number of BADLs with which caregivers had difficulty was 2.65 (SD 1.51). When caregivers who reported difficulty helping with at least one BADL were asked about the extent to which pain contributes to the difficulty participants experienced, caregivers’ average response was 6.81 (SD 1.97) on the 0–10 scale. All caregivers (100%) attributed at least some of their difficulty to pain [i.e., no caregivers reported 0 (not at all)]. The average ratings for the extent to which pain contributed to their difficulty, for each specific BADL and for the summed BADLs composite score, are shown in Table 2.

**Table 2:** Descriptive statistics.

Care tasks	Caregivers helping with activity, no. (%) (*N* = 267)	Caregivers reporting difficulty with activity, no./total (%)	Pain-attributed difficulty (0–10), mean (SD)
Basic activities of daily living			
Getting in and out of bed/chair	155 (58.1)	100/155 (64.5)	7.09 (2.20)
Eating	136 (51.0)	37/136 (27.2)	6.19 (2.28)
Showering	144 (54.0)	88/144 (61.1)	7.26 (1.96)
Dressing	132 (49.4)	45/132 (32.6)	6.80 (2.11)
Toileting	103 (38.6)	66/103 (64.1)	7.09 (2.19)
Composite of all five BADLs	200* (74.9)	127†/200 (63.5)	6.81 (1.97)
Instrumental activities of daily living			
Grocery shopping	248 (92.9)	88/248 (35.5)	6.61 (1.89)
Laundry	237 (88.8)	98/237 (41.4)	6.73 (2.07)
Cleaning	230 (86.1)	126/230 (54.8)	7.08 (1.98)
Making hot meals	229 (85.8)	83/229 (36.2)	6.13 (2.42)
Leisure	153 (57.3)	80/153 (52.3)	6.66 (2.20)
Composite of all five ADLs	263^a^ (98.5)	164^b^/263 (62.5)	6.49 (2.02)

* Represents the number of participants who helped with at least one BADL/IADL.

^†^ Represents the number of participants who reported difficulty with at least one BADL/IADL.

Similarly, of the 269 caregivers, 263 helped their care recipient with at least one IADL. Of those 263 caregivers, 164 (62%) reported difficulty with at least one IADL (Figure 1). Among caregivers reporting difficulty helping their care recipient with at least one IADL, the average number of IADLs with which caregivers had difficulty was 2.90 (SD 1.45). When caregivers who reported difficulty helping with at least one IADL were asked about the extent to which pain contributes to that difficulty, caregivers’ average response was 6.49 (SD 2.02) on the 0–10 scale. Nearly all caregivers (99%) attributed at least some of their difficulty to pain [only two caregivers reported 0 (not at all)]. The data showing the extent to which pain contributed to participants’ difficulty, on average, for each specific IADL and for the summed IADLs composite score, appear in Table 2.

### Associations between pain-attributed difficulty and caregiver demographics and caregiving characteristics

Compared to White caregivers, Black caregivers attributed less of their difficulty with BADLs to pain (estimate = –1.17, *p* = 0.04). Compared to caregivers who had been providing care for less than a year, caregivers who had been providing care for 1–2 years attributed less of their difficulty with BADLs to pain (estimate = –1.22, *p* = 0.05). There were no other significant differences in pain-attributed difficulty (Table 3). The statistical model for these estimates controlled for all other caregiver demographics and caregiving characteristics.

**Table 3:** Between-Group differences in BADL and IADL pain-attributed difficulty.

Demographics and caregiving characteristics	Average pain-attributed difficulty					
	Basic activities of daily living			Instrumental activities of daily living		
	Estimate (SE)	Contrast estimate	*p* value	Estimate (SE)	Contrast estimate	*p* value
Age						
18–39	6.48 (0.75)	[Reference]		8.05 (0.90)	[Reference]	
40–59	7.01 (0.79)	0.53	0.26	8.61 (0.93)	0.56	0.09
60+	6.35 (0.97)	−0.14	0.84	8.23 (1.01)	0.18	0.46
Gender						
Female	6.70 (0.57)	[Reference]		6.83 (0.48)	[Reference]	
Male	6.76 (0.63)	0.06	0.88	7.18 (0.53)	0.34	0.34
Other	6.38 (1.62)	−0.31	0.84	10.87 (2.32)	4.04	0.08
Race						
White	7.21 (0.63)	[Reference]		8.26 (0.82)	[Reference]	
Black	6.04 (0.85)	**−1**.**17**	0.04	7.22 (0.99)	−1.04	0.07
Asian	6.95 (1.56)	−0.26	0.80	8.44 (1.18)	0.18	0.83
Multiple Races	6.26 (1.45)	−0.94	0.45	9.26 (1.23)	1.0	0.28
Ethnicity						
Not Hispanic	6.26 (0.76)	[Reference]		8.13 (0.91)	[Reference]	
Hispanic	6.97 (0.84)	0.71	0.09	8.46 (0.92)	0.32	0.41
Income						
Under $30,000	5.96 (0.74)	[Reference]		7.68 (0.94)	[Reference]	
$30,000–$49,999	6.74 (0.87)	0.78	0.20	8.46 (0.96)	0.77	0.16
$50,000–$74,999	7.08 (0.82)	1.12	0.06	8.55 (0.93)	0.87	0.12
$75,000–$99,999	6.18 (0.99)	0.22	0.76	8.03 (1.06)	0.35	0.61
$100,000–$149,999	6.83 (0.87)	0.87	0.18	7.89 (0.93)	0.20	0.74
$150,000 or more	6.90 (1.05)	0.94	0.28	9.16 (1.12)	1.47	0.08
Relationship to care recipient						
Spouse/partner	7.19 (0.98)	[Reference]		8.92 (1.08)	[Reference]	
Child or child-in-law	6.41 (0.78)	0.79	0.23	8.23 (0.93)	−0.70 (0.59)	0.24
Related in another way	6.45 (0.94)	0.74	0.37	8.15 (8.15)	−0.77 (0.72)	0.28
Unrelated friend	6.40 (0.83)	0.79	0.34	7.90 (0.90)	−1.02 (0.80)	0.20
Presence of other caregivers						
No other caregivers	6.68 (0.80)	[Reference]		8.25 (0.92)	[Reference]	
1+ other caregivers	6.55 (0.80)	−0.13	0.74	8.34 (0.91)	0.09	0.81
Distance from care recipient						
Live in the same home	7.16 (0.72)	[Reference]		8.07 (0.83)	[Reference]	
Less than 10 miles away	6.43 (0.83)	−0.73	0.14	8.35 (0.94)	0.28	0.55
11+ miles away	6.25 (0.97)	−0.91	0.18	8.47 (1.09)	0.40	0.56
Duration of caregiving						
Less than a year	6.98 (0.83)	[Reference]		8.03 (0.93)	[Reference]	
1–2 years	5.76 (0.79)	**–1**.**22**	0.05	7.99 (0.93)	–0.04	0.95
3–5 years	6.20 (0.81)	–0.77	0.23	8.59 (0.96)	0.56	0.40
6–10 years	6.43 (1.10)	–0.55	0.55	8.36 (1.09)	0.33	0.69
More than 10 years	7.70 (1.13)	0.73	0.48	8.51 (1.14)	0.48	0.60
Hours of care per week						
20–39 h	6.90 (0.75)	[Reference]		7.81 (0.87)	[Reference]	
40–59 h	7.02 (0.77)	0.13	0.79	7.88 (0.88)	0.08	0.86
60–79 h	6.38 (0.98)	–0.52	0.46	9.07 (1.12)	1.26	0.06
80+ h	6.16 (0.94)	–0.74	0.21	8.42 (0.97)	0.62	0.24

Bolded text signifies significant difference from the reference group.

## Discussion

Recent studies suggest that a substantial number of dementia family caregivers report experiencing pain (6, 13). This study extends the literature on caregivers’ pain by indicating that, in addition to being prevalent among caregivers, pain is also potentially consequential to care outcomes. Indeed, in line with our hypothesis, caregivers in this sample not only frequently reported difficulty helping care recipients perform activities of daily living but also attributed much of that difficulty to pain. Notably, pain-attributed difficulty was high across all care tasks, even those that are less physically demanding. Physically demanding tasks such as showering and transferring received the highest pain-attributed difficulty (7.26 and 7.09, respectively), but caregivers also attributed much of their difficulty with less physical demanding care tasks to pain [e.g., helping care recipients eat (6.19)]. This finding reinforces the pervasiveness of the impact of pain on caregiving.

Notably, if the various care activities we measured in this study are performed poorly or are delayed by caregivers, care recipients’ health can be compromised (e.g., falling, hygiene issues, malnutrition), reinforcing the urgency to support caregivers’ ability to complete them fully. Supporting caregivers’ ability to perform these tasks despite pain is likely to improve care outcomes. Thus, in addition to preventing pain and injury from caregiving, mitigating the impact of caregivers’ pain on caregiving outcomes should be a priority. Practitioners could start by screening family caregivers with pain to ascertain the extent to which pain is interfering with their caregiving abilities. Also, assistive devices exist to help healthcare workers perform care tasks despite their own physical limitations (e.g., patient lifts), and this type of support should be translated to family caregiving settings.

Importantly, in this study there were minimal between-group differences in the extent to which caregivers attributed care difficulty to their pain, which was contrary to our hypotheses. Indeed, we only identified two between-group differences: Black caregivers attributed less of their difficulty to pain than White caregivers, and caregivers who had been providing care for 1–2 years attributed less of their difficulty to pain than caregivers who had been providing care for less than a year. Our finding about racial differences is in line with a recent epidemiological study of family caregivers’ pain, which found that Black caregivers were less likely to report activity-limiting pain when compared to White caregivers (6). As was speculated in that study, perhaps Black caregivers have stronger resources to aid their caregiving in ways that make physical pain less salient to caregiving (e.g., more family support). Also, despite providing more intense caregiving, Black caregivers report more positive aspects of caregiving and less emotional difficulty from caregiving compared to White caregivers, which might serve as a psychological resource that helps them better cope with pain (14). Regarding years spent caregiving, it is peculiar that the only significant difference was between caregivers who were caring for less than 1 year vs. those caring for 1–2 years. It is possible that it takes time for pain to impact caregiving, but this would not explain why there were no differences between caregivers caring for less than 1 year and, for example, caregivers who were caring for 10 or more years. Thus, we urge more scholarship on the role of time and duration of caregiving on caregivers’ pain, and the role pain plays in caregiving difficulty. Overall, however, that so few between-group differences emerged suggests that pain may universally impact caregiving difficulty for all types of caregivers.

### Strengths, limitations, and future directions

To the best of our knowledge, this is the first study to determine the extent to which dementia family caregivers with chronic pain attribute caregiving difficulty to their pain. Its nationwide reach and oversampling of Black and Hispanic caregivers are unique strengths, as is its probing of specific care tasks rather than measuring caregiving difficulty in general. Still, there are key limitations. Namely, our partnership with Qualtrics to garner a sample may have led to selection bias where certain caregivers were more likely to be recruited and to agree to complete the survey. For example, participants who willingly compete online surveys might be younger and, indeed, our sample skews younger. Though middle-aged adult-child caregivers make up a substantial proportion of dementia family caregivers across the United States, they are overrepresented in this sample. Given older caregivers are more likely to report activity-limiting pain (6), it is possible that a sample composed entirely of older caregivers would reveal even stronger pain-attributed difficulty with care tasks.

Additionally, we did not inquire about other physical (e.g., heart disease, obesity), emotional (e.g., depression, burnout) and care-recipient (e.g., dementia-related behaviors) factors that might contribute to caregiving difficulty. However, a foundational study on women's disability prompted women to report on physical symptoms that participants perceived to be the cause of their difficulty performing various activities of daily living. The study found that musculoskeletal pain was by far the most common cause of disability, even when participants were prompted with other alternative symptoms such as fatigue and balance difficulties (15). In the current study, prompting caregivers to attribute their difficulty to multiple factors, including but not limited to pain, would have offered a more complete picture of the relative role pain plays with respect to caregiving difficulty. However, the strong attribution of pain as a cause of disability in other studies as well as in the current study leads us to speculate that pain contributes significantly to the difficulty many caregivers experience helping care recipients complete various care tasks. Nonetheless, future research should ascertain factors besides pain, including other symptoms from pain (e.g., fatigue, emotional distress) that contribute to caregiver difficulty.

## Conclusion

In this nationwide study of dementia family caregivers with chronic pain, we identified pain as a substantial contributor to the difficulty caregivers experience performing care tasks. Our findings suggest that caregivers’ pain should constitute a priority for dementia care research and practice. Given the growing demand on dementia family caregivers and the rising burden of chronic pain, the number of dementia family caregivers living with chronic pain will expand. Helping this growing population manage pain better will likely reduce the difficulty caregivers experience helping care recipients perform various care tasks, which could, in turn, lead to improved caregiving outcomes.

## Data Availability

The raw data supporting the conclusions of this article will be made available by the authors, without undue reservation.

## References

[1] Alzheimer’s Association. 2024 Alzheimer’s disease facts and figures. Alzheimers Dement. (2024) 20(5):3708–21. 10.1002/alz.13809 38689398 PMC11095490

[2] Kuzuya M Enoki H Hasegawa J Izawa S Hirakawa Y Zekry D Impact of caregiver burden on adverse health outcomes in community-dwelling dependent older care recipients. Am J Geriatr Psychiatry. (2011) 19(4):382–91. 10.1097/JGP.0b013e3181e9b98d 20808120

[3] Sullivan SS de Rosa C Li CS Chang YP. Dementia caregiver burdens predict overnight hospitalization and hospice utilization. Palliat Support Care. (2023) 21(6):1001–15. 10.1017/S1478951522001249 36263744 PMC10115915

[4] Pristavec T Luth EA. Informal caregiver burden, benefits, and older adult mortality: a survival analysis. J Gerontol B Psychol Sci Soc Sci. (2020) 75(10):2193–206. 10.1093/geronb/gbaa001 31903481 PMC7664316

[5] Schulz R Beach SR Friedman EM. Caregiving factors as predictors of care recipient mortality. Am J Geriatr Psychiatry. (2021) 29(3):295–303. 10.1016/j.jagp.2020.06.025 32718853 PMC7782207

[6] Turner SG Robinson JRM Pillemer KA Reid MC. Prevalence estimates of arthritis and activity-limiting pain among family caregivers to older adults. Gerontologist. (2024) 64(5):gnad124. 10.1093/geront/gnad124 37656675 PMC11020308

[7] Moon H Dilworth-Anderson P. Baby boomer caregiver and dementia caregiving: findings from the national study of caregiving. Age Ageing. (2015) 44(2):300–6. 10.1093/ageing/afu119 25359299 PMC4339725

[8] Beach SR Schulz R. Family caregiver factors associated with unmet needs for care of older adults. J Am Geriatr Soc. (2017) 65(3):560–6. 10.1111/jgs.14547 27935019

[9] Turner SG Pillemer K Demetres M Ettinger W Reid MC Zlotnick C Physical pain among family caregivers to older adults: a scoping review of the literature. J Am Geriatr Soc. (2024) 72(9):2853–65. 10.1111/jgs.19037 38895995 PMC11368645

[10] Rikard SM Strahan AE Schmit KM Guy GP Jr. Chronic pain among adults—United States, 2019–2021. MMWR Morb Mortal Wkly Rep. (2023) 72:379–85. 10.15585/mmwr.mm7215a1 37053114 PMC10121254

[11] Moss AJ Hauser DJ Rosenzweig C Jaffe S Robinson J Litman L. Using market-research panels for behavioral science: an overview and tutorial. Adv Methods Pract Psychol Sci. (2023) 6(2):25152459221140388. 10.1177/25152459221140388

[12] Turner SG Witzel DD Garza S Pillemer K Reid MC. Health patterns of dementia caregivers with chronic pain: latent profile analysis of the PROMIS-29 measure. J Appl Gerontol. (2025) 13:07334648251360819. 10.1177/07334648251360819 PMC12548739 40738141

[13] Turner SG Reid MC Pillemer KA. Pain prevalence and intensity among family caregivers versus non-caregivers in the United States. J Aging Health. (2025). 10.1177/08982643251331247 PMC12353421 40205876

[14] Fabius CD Wolff JL Kasper JD. Race differences in characteristics and experiences of black and white caregivers of older Americans. Gerontologist. (2020) 60(7):1244–53. 10.1093/geront/gnaa042 32400881 PMC7491434

[15] Leveille SG Fried L Guralnik JM. Disabling symptoms. J Gen Intern Med. (2002) 17:766–73. 10.1046/j.1525-1497.2002.20229.x 12390552 PMC1495119

