# Supplementary material for: Pain-attributed care task difficulty among dementia caregivers with chronic pain
Source: Front Pain Res (Lausanne). 2025 Nov 5;6:1661457. doi: 10.3389/fpain.2025.1661457 (PMC12627032; doi:10.3389/fpain.2025.1661457)

Supplemental Materials

Histograms and Q-Q plots for Pain-Attributed Difficulty with Basic Activities of Daily Living (BADLs) and Instrumental Activities of Daily Living (IADLs)

A. Histogram Pain-Attributed Difficulty with Basic Activities of Daily Living (BADLs)

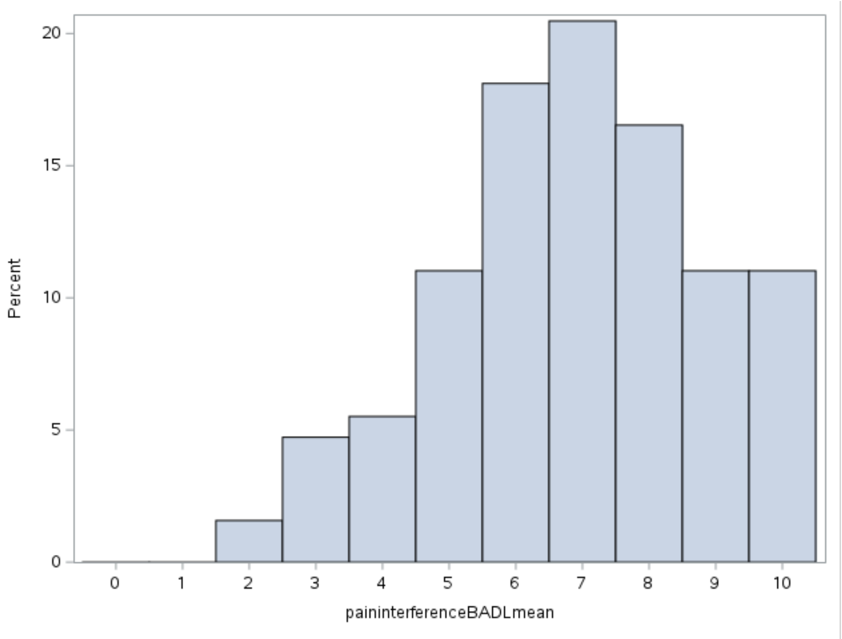

B. Histogram Pain-Attributed Difficulty with Instrumental Activities of Daily Living (IADLs)

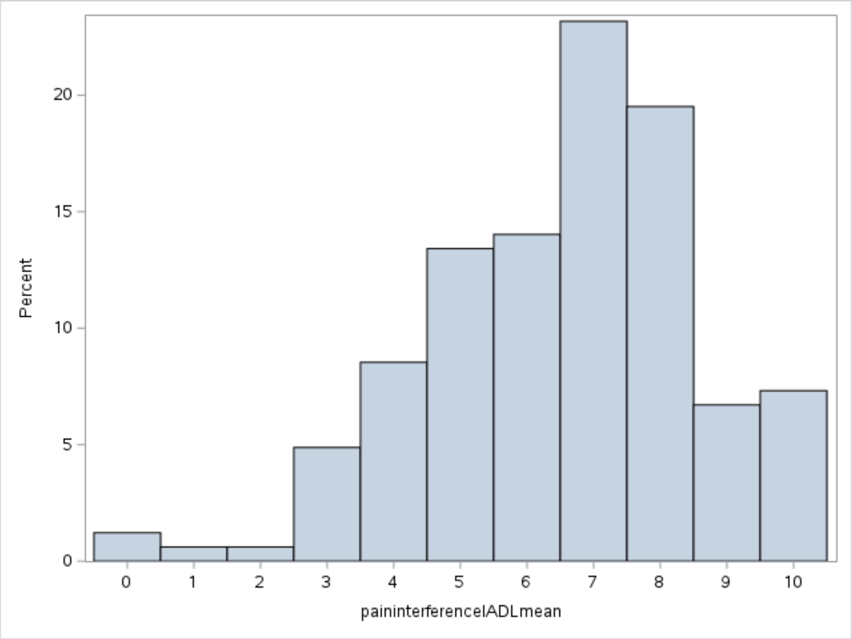

C. Q-Q Plot: Pain-Attributed Difficulty with Basic Activities of Daily Living (BADLs)

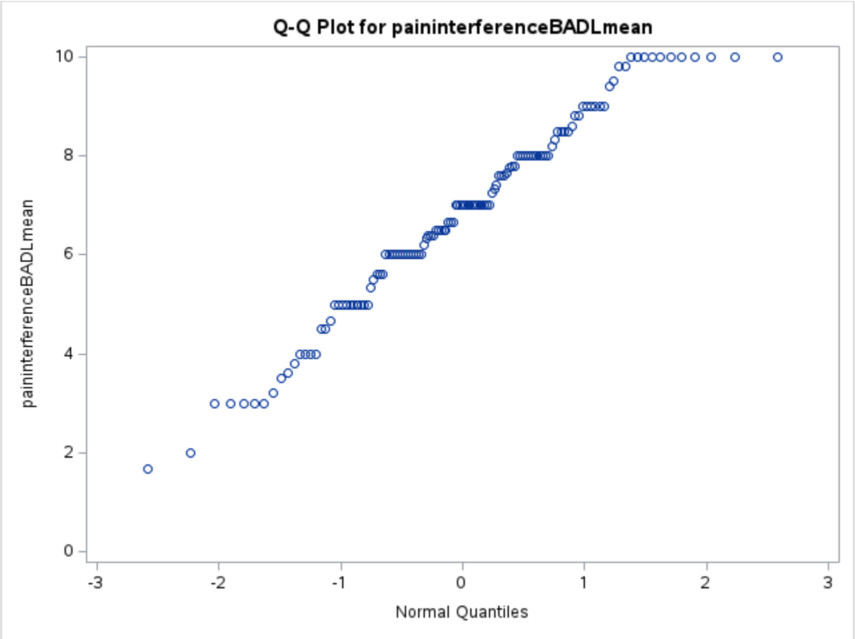

D. Q-Q Plot: Pain-Attributed Difficulty with Instrumental Activities of Daily Living (IADLs)

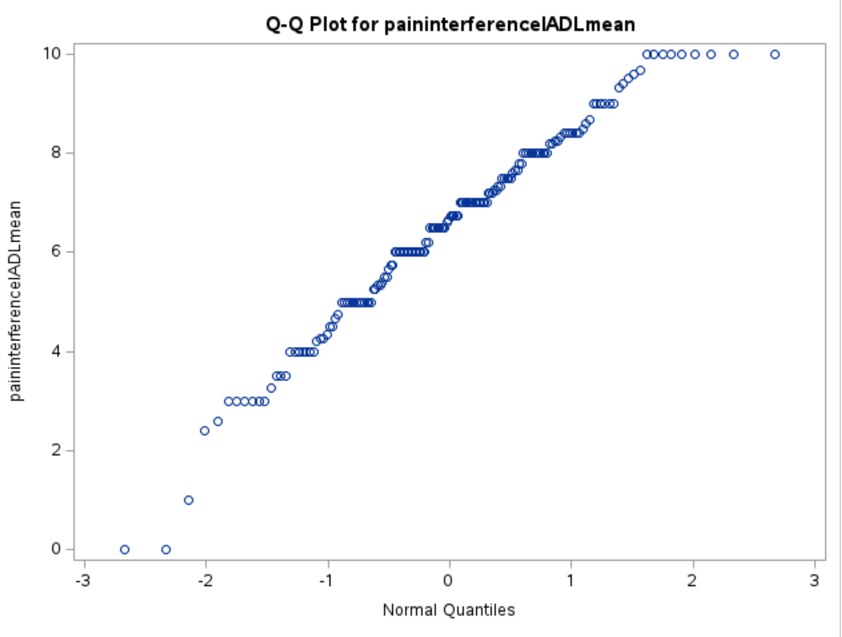

Supplement: Supplementary file 1 [file Datasheet1.pdf]
